# Protective effect of baicalin on oxidative stress injury in retinal ganglion cells through the JAK/STAT signaling pathway *in vitro* and *in vivo*

**DOI:** 10.3389/fphar.2024.1443472

**Published:** 2024-10-31

**Authors:** Huan Yu, Dan Zhou, Wei Wang, Qingxia Wang, Min Li, Xiaoyun Ma

**Affiliations:** ^1^ Graduate School of Shanghai University of Traditional Chinese Medicine, Shanghai, China; ^2^ Zhoupu Hospital, Shanghai University of Medicine and Health Sciences, Shanghai, China; ^3^ Department of Ophthalmology, Shanghai Tenth People’s Hospital, Tongji University, Shanghai, China

**Keywords:** baicalin, retinal ganglion cells, oxidative stress, JAK/STAT signaling pathway, inflammatory, apoptosis

## Abstract

**Background and Purpose:**

The damage or apoptosis of retinal ganglion cells (RGCs) is one of the leading causes of various blinding eye diseases, such as glaucoma, diabetic retinopathy, optic neuritis, and ischemic optic neuropathy. Oxidative stress is involved in RGCs death. Baicalin, a flavonoid compound extracted from Scutellaria baicalensis, has various beneficial effects, including anti-inflammatory, anti-apoptotic, and antioxidant properties. However, the effects of baicalin on RGCs and the underlying mechanisms require further investigation.

**Methods:**

In this study, a glutamate-induced oxidative stress damage model of R28 cells and a rat retinal injury model were established to investigate the effects of baicalin on oxidative stress damage to RGCs and try to elucidate the underlying mechanism.

**Results:**

*In vitro* experiments demonstrated that the survival rate of R28 cells after glutamate treatment dropped to 33.4%, while 10 μM baicalin significantly inhibited glutamate-induced damage in RGCs (*P* < 0.001) and enhanced cell viability through decreasing ROS levels, increasing antioxidant enzyme activity, and suppressing the expression of inflammatory factors iNOS, TNF-α, IL-6, and IL-1β (*P* < 0.001). *In vivo*, baicalin effectively mitigated structural damage to retinal tissue and RGCs morphology induced by glutamate, increasing the thickness of the retinal ganglion cell layer, improving RGCs density, and reducing overall retinal thinning in rats (*P <* 0.001) in a time- and dose-dependent effects. Mechanistic studies revealed that glutamate evaluated the phosphorylation levels of JAK/STAT, while baicalin effectively inhibited the activation of the JAK/STAT signaling pathway.

**Conclusion:**

This study confirmed that baicalin protects against glutamate-induced oxidative stress damage in RGCs. It effectively alleviates oxidative stress and inflammatory responses, reduces cell apoptosis, and improves the pathological changes in the retina of rat models of RGCs damage, thereby decreasing RGCs death. Further exploration of its mechanism revealed that baicalin effectively inhibits the JAK/STAT signaling pathway, protecting RGCs from oxidative stress damage. This provides an experimental basis for the application of baicalin in the treatment of RGCs damage.

## 1 Introduction

The damage or apoptosis of retinal ganglion cells (RGCs) is one of the leading causes of blinding eye diseases such as glaucoma, diabetic retinopathy, optic neuritis, and ischemic optic neuropathy (Chen et al., 2022; Kang et al., 2021; Zhu et al., 2018). Various factors, including oxidative stress, aging, glutamate neurotoxicity, and susceptibility genes, influence the occurrence and progression of these eye diseases. The primary pathological damage involves the progressive injury of RGCs and vision loss (Wong and Benowitz, 2022). Therefore, strengthening the prevention and treatment of eye diseases related to RGCs damage is essential (Tham et al., 2014; Tan and Wong, 2023).

RGCs belong to a type of nerve cell located in the neural epithelial layer of the retina. They are the only cells that transmit visual signals to the brain to form images (Gu et al., 2022), requiring a large amount of adenosine triphosphate to support the energy needed for frequent transmission of visual stimuli (Yu et al., 2013). In the energy generation process, the electron transport chain in mitochondria forms a large amount of ROS. At the same time, in the human eye tissue, there are already antioxidant substances such as superoxide dismutase (SOD) and glutathione (GSH) (Ji and Yeo, 2021) to keep the intracellular environment stable. However, once this process is disrupted, and many oxidants appear or antioxidants are insufficient, it will cause an imbalance between the generation and consumption of ROS, thereby causing oxidative stress damage (Pisoschi et al., 2021; Sacca et al., 2015; Molayi-jabdaragi et al., 2020; Youle and Van Der Bliek, 2012), ultimately leading to the death of RGCs.

Baicalin, a flavonoid isolated from *scutellaria baicalensis*, has anti-inflammatory, antioxidant, anti-apoptosis, and other effects (Zhu et al., 2020; Fan et al., 2021; Sui et al., 2021) and is vital in treating glaucoma, cataract, and other eye diseases. For example, it can protect human trabecular reticulum cells from oxidative stress (Gong and Zhu, 2018) and improve the pathological changes of the retinal nerve fiber layer in glaucoma (Yang et al., 2021). However, the effect of baicalin on RGCs oxidative damage and its mechanism need to be further studied.

The JAK/STAT signaling pathway can transfer signals of various cytokines and growth factors to the nucleus, regulate gene expression, and affect cell proliferation, differentiation, migration, apoptosis, immune response, and other cellular processes (Hu et al., 2021; Rusek et al., 2023). Studies have shown that miR-216a can protect human retinal microvascular endothelial cells from damage in diabetic retinopathy by inhibiting the NOS2/JAK/STAT pathway (Liu et al., 2020). Gypenosides can inhibit the overexpression of STAT3 and JAK2 mRNA in the retina, thereby inhibiting the activation of astrocytes and delaying the apoptosis of RGCs (Wang et al., 2022). In addition, IL-6, a primary activator of the JAK/STAT signaling pathway, was detected in the aqueous humor of glaucoma patients (Ahmad and Ahsan, 2020). Therefore, the above study suggests that the JAK/STAT signaling pathway may be involved in regulating RGCs oxidative damage.

Therefore, this study aims to investigate the protective mechanism of baicalin on oxidative stress damage of RGCs induced by glutamate *In vitro* and *in vivo* by measuring the morphological changes of RGCs, the expression changes of apoptosis, inflammation, and oxidative stress-related proteins, and the activation of JAK/STAT signaling pathway.

## 2 Materials and methods

### 2.1 Reagents

Glutamic acid (Glu, Aladdin, Shanghai, China) was added to a 1 M HCl solution and heated at 100°C in a water bath for 1 h to ensure complete dissolution of the glutamic acid. Baicalin (BAI, #572667, Sigma-Aldrich, Germany, purity: 95%, solubility: ≥100 mg/mL in DMSO) was added to a DMSO solution to achieve complete dissolution of the baicalin, and the baicalin solution was prepared fresh for immediate use (Diagram 1).

### 2.2 Cell culture

The R28 retinal precursor cell line is an immortalized adherent retinal precursor cell line derived from the retinas of Sprague-Dawley rats. The cells were obtained from 6-day-old female Sprague-Dawley rats and were immortalized using the psi2 replication-incompetent retroviral vector. This cell line has been characterized and utilized in various *In vitro* and *in vivo* studies of retinal cells, including differentiation, neuroprotection, cytotoxicity, as well as retinal gene expression and neuronal function, specifically for *In vitro* studies of neuroprotection, cytotoxicity, and physiological functions of RGCs (Gm, 2014). The R28 cells were cultured in low-glucose DMEM (Gibco, Waltham, Massachusetts, United States) supplemented with 10% FBS (Gibco, NY, United States) and 1% penicillin/streptomycin and were maintained in a humidified incubator at 37°C with 5% CO_2_. The R28 retinal precursor cell line is an immortal adherent cell line derived from the retina of Sprague-Dawley rats. R28 cells were cultured in low-glucose DMEM (Gibco, Waltham, Massachusetts, United States) supplemented with 10% FBS (FBS, Gibco, NY, United States) and 1% penicillin/streptomycin (Gibco, Waltham, Massachusetts, United States). The cells were cultured in a humidified incubator at 37°C with 5% CO_2_.

### 2.3 Animals

Eleven-week-old male Sprague-Dawley rats (450 g ± 20 g) (Jihui Experimental Animal Co., Shanghai, China.) were housed in rat cages under a 12 h light/dark cycle, with a temperature of 21°C ± 2°C and a humidity of 50% ± 5%. Water and food were provided *ad libitum*. All animal experiments were conducted in accordance with the ethical guidelines of the Association for Research in Vision and Ophthalmology for the use of animals in research (Ethics Number: 2024-C-084-E01).

### 2.4 Cell damage model

R28 cells were seeded in a 96-well cell culture plate at a density of 7×10^3^ cells per well. Once the cell growth status and density reached 80%–90%, 5 mM glutamate was added to induce oxidative stress damage in R28 cells. The cells were then incubated in a 37°C constant temperature CO_2_ incubator for 24 h. After incubation, the cells were washed twice with PBS, and different concentrations of baicalin (1 μM, 5 μM, 10 μM, 15 μM, 20 μM, 50 μM, and 100 μM) were added. The cells were subsequently incubated in a 37°C constant temperature CO_2_ incubator.

### 2.5 Animal models establishment and treatment

The rats were randomly divided into six groups, with nine animals in each group: a regular control group, a glutamate injury group, a DMSO group, a low-dose baicalin group (10 mg/kg), a medium-dose baicalin group (20 mg/kg), and a high-dose baicalin group (30 mg/kg). Each group underwent three independent *in vivo* biological experiments. Intraperitoneal injection of 1.5% pentobarbital sodium (30 mg/kg) was used to ensure that the rats were under sufficient anesthesia to expose the right eye. A 30G puncture needle was inserted into the vitreous cavity at a location 0.5–1.0 mm posterior to the temporal limbus. A slow injection of 5 μL of 30 mM glutamate was performed (Ma et al., 2019). After 24 h, the DMSO and the treatment groups were administered 10% DMSO and low, medium, and high concentrations of baicalin (10, 20, and 30 mg/kg) via tail vein injection once daily for 14 consecutive days (Gao et al., 2021). Tests and samples were taken at 3, 7, and 14 days of administration (Diagram 2).

### 2.6 Cell viability

The effects of glutamate and chloroquine on R28 cell viability were assessed using a CCK-8 (Beyotime, Jiangsu, China) assay. R28 cells were seeded at 7000 cells per well in a 96-well plate and cultured for 24 h. Subsequently, the cells were treated with specified doses of glutamate for a specified duration. Following treatment, 10 μL of 5 mg/mL CCK-8 reagent was added to each well containing 100 μL of medium, and the cells were then incubated in a humidified incubator at 37°C for 2 h. The absorbance of the cells at a wavelength of 450 nm was measured using an enzyme-labelling measuring instrument. The cell viability in each group is calculated as follows: cell viability (%) = [absorbance of experimental wells - absorbance of blank wells)/(absorbance of control wells - absorbance of blank wells)] × 100%.

### 2.7 Hematoxylin and eosin (HE) staining

After glutamate injection for 3, 7, and 14 days, rats were euthanized by intraperitoneal injection of an excessive amount of pentobarbital. The eyeballs were then removed and fixed with 4% paraformaldehyde, dehydrated with gradient alcohol, and embedded in paraffin. Subsequently, samples were cut into 5 μm thick sections at the optic disc after paraffin embedding. Then, it was stained with an H&E solution using a Hematoxylin-Eosin staining kit (Solarbio, Beijing, China). After clearing, all sections were sealed with neutral resin, and images of the samples were captured using an optical microscope (Leica, Wetzlar, Germany). Additionally, the number of retinal ganglion cells within a distance of 300–750 μm from the optic disc was manually counted.

### 2.8 Optical coherence tomography (OCT) measurement

After anesthetizing the rats and dilating the pupils, OCT (Topcon, Japan) was utilized to examine all rats. The animals were positioned on a lifting platform to adjust eye position, ensuring that the optic nerve was centered in the image and the retinal image was clear. The retinal thickness was measured at 1000–2000 μm from the center of the optic disc head. Two skilled operators took and analyzed measurements, and the average value was derived from the combined measurements.

### 2.9 ROS detection

DCFH-DA (Beyotime, Jiangsu, China) was diluted in serum-free culture medium at a ratio of 1:1000 to achieve a final concentration of 10 μM. The culture medium was removed from each well of the 96-well plate, and the wells were washed twice with PBS. Then, 100 μL of the diluted DCFH-DA solution was added to each well and incubated in a dark environment at 37°C for 20 min to allow DCFH-DA to enter the cells and be metabolized to DCF within the cells. Subsequently, the cells were washed three times with a serum-free culture medium to remove any unincorporated DCFH-DA. Fluorescence was observed using an inverted fluorescence microscope to identify fluorescent wells, after which DAPI (Beyotime, Jiangsu, China) was added for continued observation and imaging. The fluorescence intensity was analyzed using ImageJ software. Calculation method: ROS detection value/CCK-8 detection value of the corresponding hole.

### 2.10 SOD activity detection

The WST-8/enzyme working solution and reaction initiation working solution were prepared according to the instructions of the SOD assay kit (Beyotime, Jiangsu, China). Samples from each group were mixed with the WST-8/enzyme working solution, with 160 μL of the mixture for each sample. The mixture was then placed into a 96-well plate, with 160 μL added to each well. Subsequently, 20 μL of the reaction initiation working solution was added to each well to initiate the reaction. After incubating at 37°C for 30 min, the absorbance was measured at 450 nm, and the readings were adjusted by subtracting the absorbance values at the reference wavelength to obtain the actual measured values.

### 2.11 Glutathione detection

The total glutathione assay working solution was prepared according to the instructions of the GSH assay kit (Sigma, CS0260). Cells in a 6-well plate were digested, and the supernatant was discarded after centrifugation. A volume of three times the cell pellet was added to the prepared 5% protein removal reagent S, and the mixture was thoroughly vortexed. The samples were subjected to two rapid freeze-thaw cycles using liquid nitrogen and a 37°C water bath. The supernatant was then collected by centrifugation, and the absorbance at 412 nm was immediately measured using a microplate reader, with measurements taken every 5 min for a total duration of 25 min.

### 2.12 Western blot analysis

*In vitro* experiment, lysis of R28 cells was performed using RIPA lysis buffer (Beyotime, P0013B), followed by incubation of the cells on ice for 30 min. The supernatant was collected after centrifugation of the cell lysate at 4°C and 400 g for 20 min. *In vivo* experiments, the retina of the right eye of the rats was taken on the third, seventh, and 14th day and dissected under the microscope. Subsequently, 100 μL of lysis buffer was added to the retinal tissue, and the tissue was centrifuged at 4°C and 10,000 g for 15 min. Finally, the supernatant was transferred to a new centrifuge tube. Protein concentration was determined using the BCA protein assay kit (Beyotime, P0012). A total of 40 µg of protein was subjected to 10% SDS-PAGE and transferred onto a PVDF membrane (Beyotime, FFP28), followed by incubation with 5% skim milk for 4 h. The membrane was then incubated with primary antibodies overnight at 4°C. The primary antibodies used were as follows: B-cell lymphoma 2(Bcl-2) antibody (Abcam, ab182858), Bcl-2-associated X protein (Bax) antibody (Abcam, ab32503), Neuron-specific class III beta-tubulin (Tuj-1) antibody (Abcam, ab52623), Inducible Nitric Oxide Synthase (iNOS) antibody (Abcam, ab178945), Tumor Necrosis Factor-alpha (TNF-α) antibody (Abcam, ab205587), Interleukin-6(IL-6) antibody (Abcam, ab259341), Interleukin-1 beta (IL-1β) antibody (Abcam, ab254360), p-JAK1 antibody (Abcam, ab278781), JAK1 antibody (Cell Signaling, 3344T), p-JAK2 antibody (Abcam, ab32101), JAK2 antibody (Abcam, ab108596), p-STAT1 antibody (Abcam, ab109461), STAT1 antibody (CST, mAb #14994), p-STAT2 antibody(Abcam, ab0284), STAT2 antibody (Abcam, ab32367), p-STAT3 antibody(CST, mAb #9145) and STAT3 antibody (Abcam, ab68153), The PVDF membrane was subsequently incubated with goat anti-rabbit IgG secondary antibody (1:700; Sigma) at room temperature for 2 h. Finally, the PVDF membrane was visualized using HRP horseradish peroxidase (1:1,000, Beyotime, Shanghai, China), and the protein band density was quantified using ImageJ software.

### 2.13 Real-time Quantitative polymerase chain reaction (RT-qPCR)

The R28 cells were lysed in Trizol Reagent, after which one-fifth volume of chloroform was added. The mixture was then centrifuged at 13,000×rpm for 15 min at 4°C. The aqueous phase was mixed with an equal volume of 100% isopropanol and incubated for 10 min at room temperature. RNA was precipitated by centrifugation at 13,000×rpm for 10 min at 4°C. The RNA pellet was washed with 70% RNase-free ethanol and dissolved in RNase-free H2O. One microgram of total RNA was used for reverse transcription using Expand reverse transcriptase (Roche, United States) at 42°C for 1 h in a total volume of 20 μL. Equal amounts of cDNAs were then PCR-amplified using appropriate primers. The primer sequences are provided in Table 1.

**TABLE 1:** Primers sequences.

Genes	Forward primer	Reverse primer
iNOS	GAA​GGA​GAT​GAG​GAA​CAG​CG	CAG​GAG​TTG​GCT​CAC​AGT​CA
TNF-α	ATGGGCTCC CTCTCATCAGT	GCTTGGTGGTTT GCTACGAC
IL-6	CCAGTTGCCTT CTTGGGACT	TGCCATTGCACAAC TCTTTTC
IL-1β	TCA​TCT​TTG​AAG​AAG​AGC​CCG	TCAGACAGCAC GAGGCATTT
Tuj-1	GTG​AGT​TCC​TCT​TTG​CCT​TCT	GGC​TGG​ACC​TGC​ATC​TTT​AT
Bax	GCG​ATG​AAC​TGG​ACA​ACA​AC	GAT​CAG​CTC​GGG​CAC​TTT​A
BCL-2	CTG​GCT​TGA​CTG​GCT​GAA​TA	GTT​CCC​TCC​GAA​CGG​TAA​AT
GAPDH	TCCAAC CCAACCCTCAACAG	CCGATACG GCCAAATCCGTT

### 2.14 Statistical analysis

The measurement data in this experiment were expressed as mean ± standard deviation and collected in ≥3 independent experiments. ImageJ software analyzed the gray values and fluorescence intensities of the strips obtained from Western blot and fluorescent microscope analyses. Data plotting was performed using GraphPad Prism 9.0 (GraphPad Software, La Jolla, California, United States), and data analysis was conducted using SPSS 26.0 (IBM, Armonk, NY, United States) statistical software. The Student’s t-test was used to compare the two groups of data. Multi-group comparisons were performed using one-way analysis of variance (ANOVA), followed by Turkey’s multiple comparisons when comparing more than two groups, with statistical significance at *p* < 0.05.

## 3 Results

### 3.1 Protective effect of baicalin on the glutamate-induced RGCs injury

**FIGURE 1:**
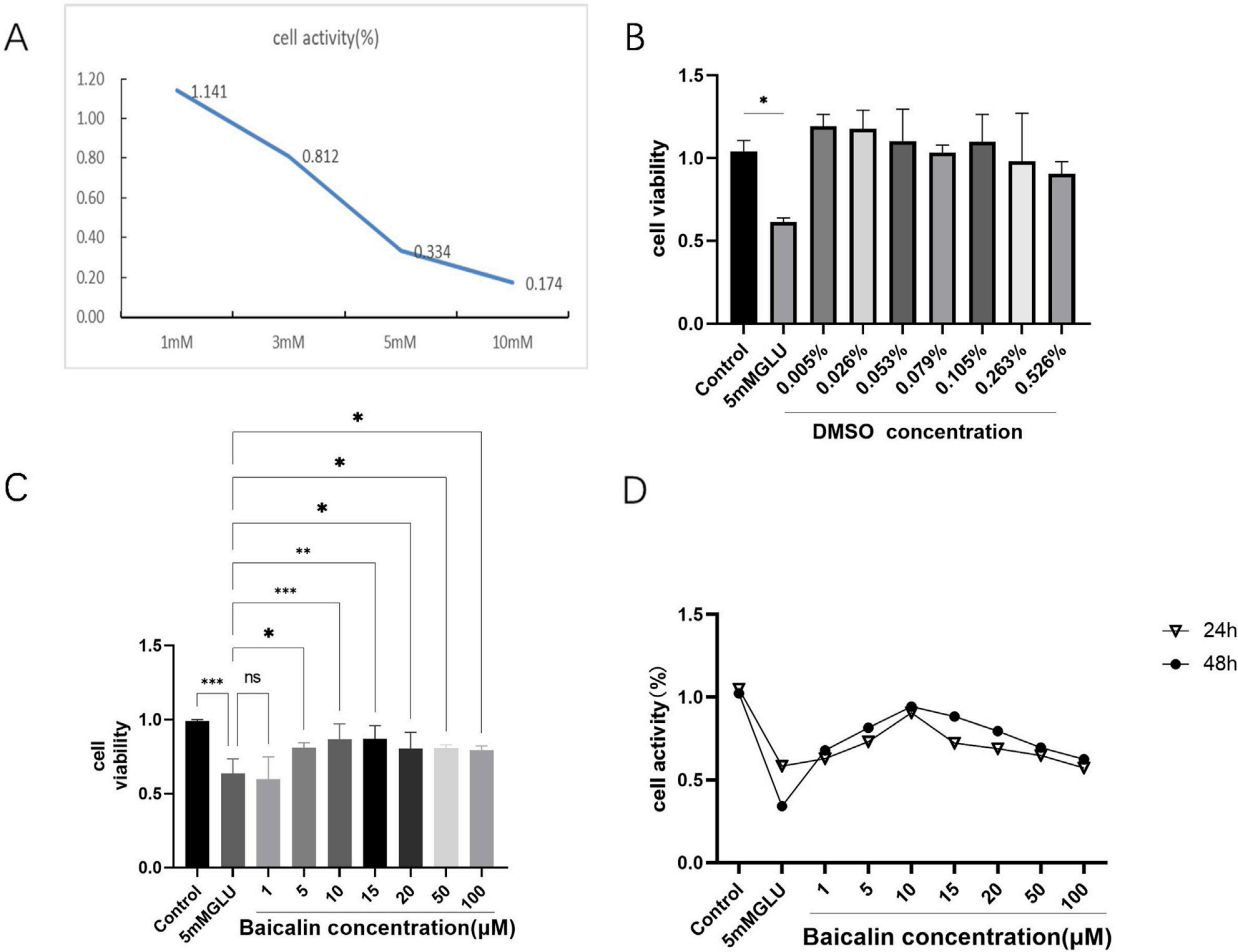
Baicalin improved the viability of glutamate-induced R28 cell injury. **(A)** Oxidative-stress damage of R28 cells induced by different concentrations of glutamate (1mM, 3mM, 5mM, and 10 mM) for 24 h. **(B)** Effect of dimethyl sulfoxide (DMSO) concentrations corresponding to baicalin at different concentrations on the cell viability of R28 cells after 24 h **(C, D)** The viability of R28 cells treated with various concentrations of baicalin (1 μM, 5 μM, 10 μM, 15μM, 20 μM, 50 μM, and 100 μM) for 24 and 48 h. Data represent the mean ± SEM of three independent experiments. *, *p <* 0.05; **, *p <* 0.01; ***, *p <* 0.001. indicate significant differences and ns > 0.05 means no significant difference.

Immortalized rat retinal progenitor cells (R28) were treated with 1 mM, 3 mM, 5 mM, and 10 mM glutamate for 24h. Cell viability significantly decreased in a concentration-dependent manner, as shown in Figure 1A. The activity of R28 cells was minimally affected by the 1 mM glutamate treatment. However, with increasing concentrations of glutamate to 3 mM and 5 mM, a substantial reduction in R28 cell activity was observed, with cell survival rates of 81.2% and 33.4%, respectively. These rates indicate a significant decrease in cell activity compared to normal levels. A more pronounced decrease in cell activity was noted with the 10 mM glutamate treatment, where the survival rate dropped to only 17.4%, demonstrating the strong toxic effect of high glutamate concentrations on R28 cells. Consequently, the oxidative damage model for R28 cells was established in subsequent experiments by treating them with 5 mM glutamate for 24 h.

To determine the protective effect of baicalin, the effect of its solvent DMSO on cells was first detected by CCK-8 assay, considering its water insolubility. The results showed that the concentration of DMSO required in the experiment did not affect R28 cells (Figure 1B), further supporting the potential application of baicalin. Subsequently, 24 h after glutamic acid injury, R28 cells were treated with baicalin at different concentrations (1 μM, 5 μM, 10 μM, 15 μM, 20 μM, 50 μM, and 100 μM) for 24 and 48 h, respectively. The CCK-8 detection results showed (Figure 1C) that R28 cells were damaged by 5 mM glutamic acid. Little effect on cell activity was observed with low concentration baicalin (1 μM), and as the concentration increased, cell activity began to increase. The most significant protective effect on glutamate-induced R28 injury was observed with 10 μM baicalin (*p <* 0.001). However, with the continuous increase in concentration, cell activity began to decline, suggesting a decrease in the protective effect of baicalin. Figure 1D compares the changes in cell activity after treatment with baicalin at different concentrations for 24 and 48 h. The results showed that R28 cells were damaged by 5 mM glutamic acid, and the cell viability fluctuated slightly after treatment with DMSO at different concentrations for 24 and 48 h, with no significant difference (*p* > 0.05). It is suggested that the DMSO required in this study does not cause damage to cells or affect the effect of baicalin. A comprehensive analysis revealed that baicalin concentration significantly affected cell viability when 5 mM glutamic acid damaged R28 cells, with 10 μM baicalin showing a significant protective effect. However, the protective effect of baicalin gradually weakened with increasing concentration.

### 3.2 Baicalin inhibits the apoptosis of RGCs induced by glutamate

**FIGURE 2:**
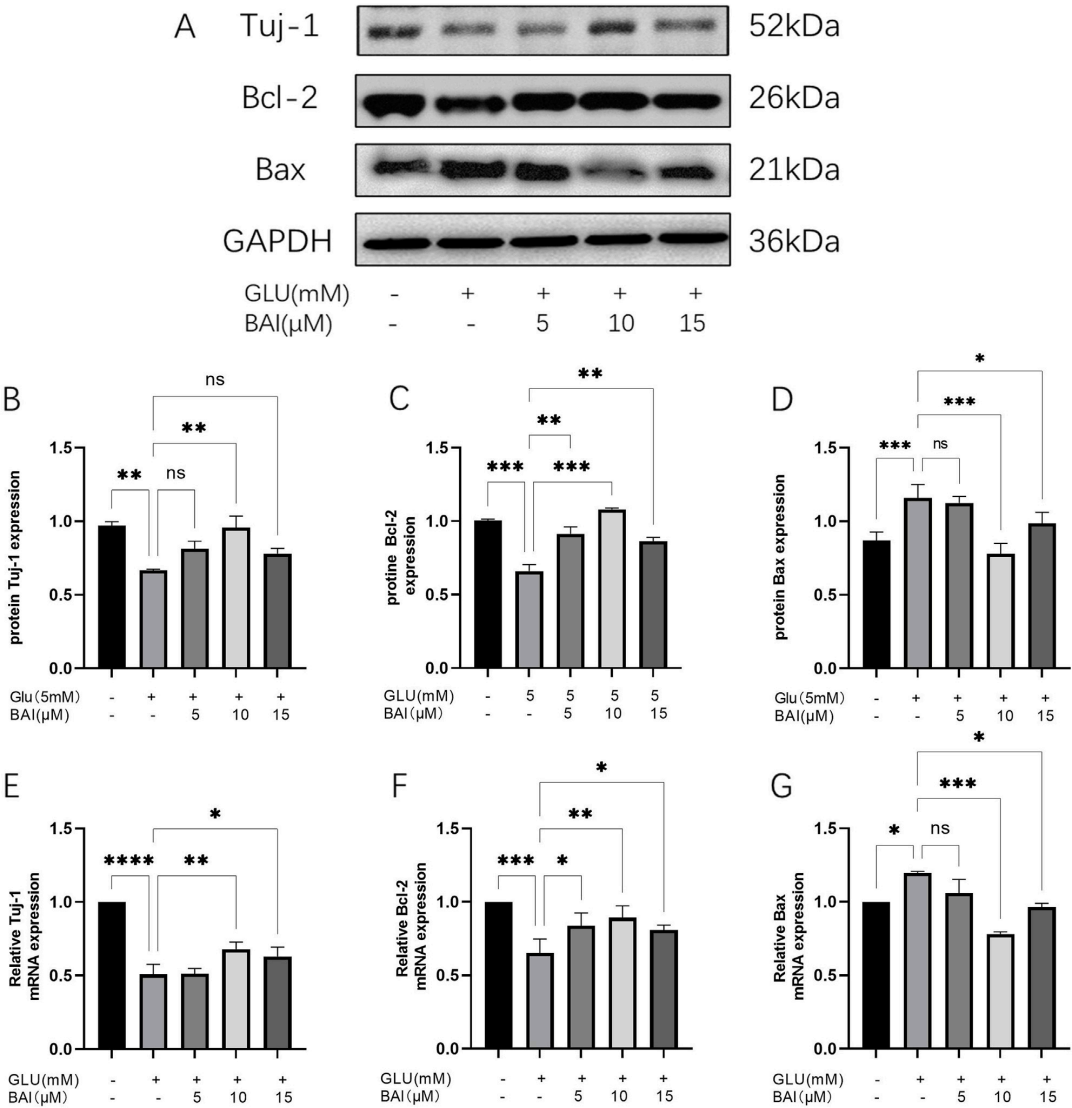
Baicalin inhibited glutamate-induced R28 cell apoptosis. **(A–D)** The protein expression of Tuj-1, Bcl-2, and Bax was detected by Western blot. **(E–G)** The mRNA expression of Tuj-1, Bcl-2, and Bax was detected by qRT-PCR. Data represent the mean ± SEM of three independent experiments. *, *p <* 0.05; **, *p <* 0.01; ***, *p <* 0.001 indicate significant differences, and *ns* > 0.05 means no significance difference.

To further determine the safe concentration and protective effect of baicalin, baicalin concentrations of 5 μM, 10 μM, and 15 μM were selected to detect its anti-apoptotic effect. Western blot analysis showed that (Figure 2A), under glutamate stimulation, upregulation of Bax in R28 cells was observed (Figure 2D, *p <* 0.001), while the downregulation of the RGCs marker Tuj-1 (Figure 2B, *p* = 0.0036) and the anti-apoptotic protein Bcl-2 (Figure 2C, *p <* 0.001) indicated that glutamate treatment induced apoptosis. However, after baicalin treatment, the expression of these proteins was reversed, suggesting that baicalin might inhibit glutamate-induced apoptosis of R28 cells by regulating the expression of these proteins, with the anti-apoptotic effect of baicalin at 10 μM showing more significant results (*p* = 0.0043, *p <* 0.001). Additionally, the results of RT-qPCR (Figures 2E, F) were consistent with the Western blot results. After baicalin treatment with a concentration of 10 μM, the mRNA expression of Tuj-1 and Bcl-2 was significantly increased compared with the glutamate group (*p* = 0.0018, *p* = 0.0033), whereas the mRNA expression of Bax was decreased (*p <* 0.001), further supporting the regulatory effect of baicalin on apoptosis.

### 3.3 Baicalin can inhibit glutamate-induced oxidative stress injury of R28 cells

**FIGURE 3:**
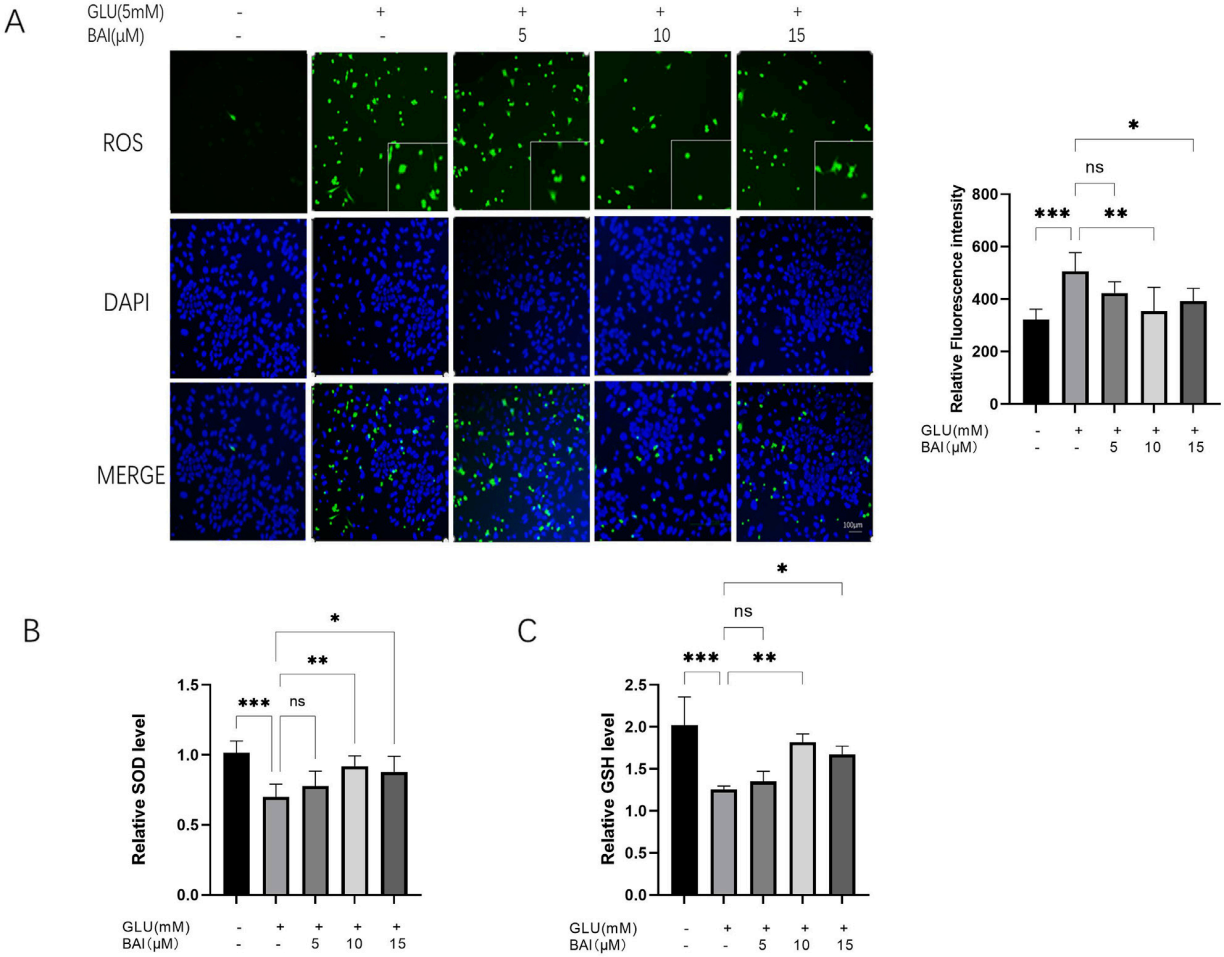
Baicalin inhibits glutamate-induced oxidative stress damage. **(A)** Representative images of reactive oxygen species (ROS) fluorescence and fluorescence intensity analysis of glutamate-damaged R28 cells treated with different concentrations of baicalin (5 μM, 10 μM, and 15 μM). **(B, C)** Effects of baicalin concentrations (5 μM, 10 μM, and 15 μM) on superoxide dismutase (SOD) and glutathione (GSH) levels in R28 cells induced by 5 mM glutamate. Data represent the mean ± SEM of three independent experiments. *, *p <* 0.05; **, *p <* 0.01; ***, *p <* 0.001 indicate significant differences, and *ns* > 0.05 means no significance difference.

Oxidative stress injury can increase ROS levels. Previous studies have indicated that oxidative stress, induced by glutamate, can stimulate the production of ROS and inflammatory factors, thereby promoting neuronal cell death (Zhu et al., 2020; Fan et al., 2021). Our study demonstrated that exposure of R28 cells to glutamate led to a significant increase in ROS production (Figures 3A, B), consistent with the detrimental effects of glutamate-induced oxidative stress. However, treatment with 10 μM baicalin significantly reduced the green fluorescence signal intensity of ROS (Figure 3A) and decreased ROS levels (Figure 3B, *P <* 0.001), suggesting that baicalin may mitigate oxidative stress damage caused by glutamate. Further evaluation of the oxidative stress and antioxidant capacity of cells revealed that glutamate-induced ROS levels increased (*p <* 0.001), whereas activities of superoxide dismutase (SOD) and glutathione (GSH) decreased in R28 cells (Figures 3C, D, P < 0.001). These alterations were significantly ameliorated following baicalin treatment (*p* = 0.0093, *p* = 0.0033), highlighting the potent antioxidant effect of 10 μM baicalin.

### 3.4 Baicalin can inhibit glutamate-induced inflammation in R28 cells

**FIGURE 4:**
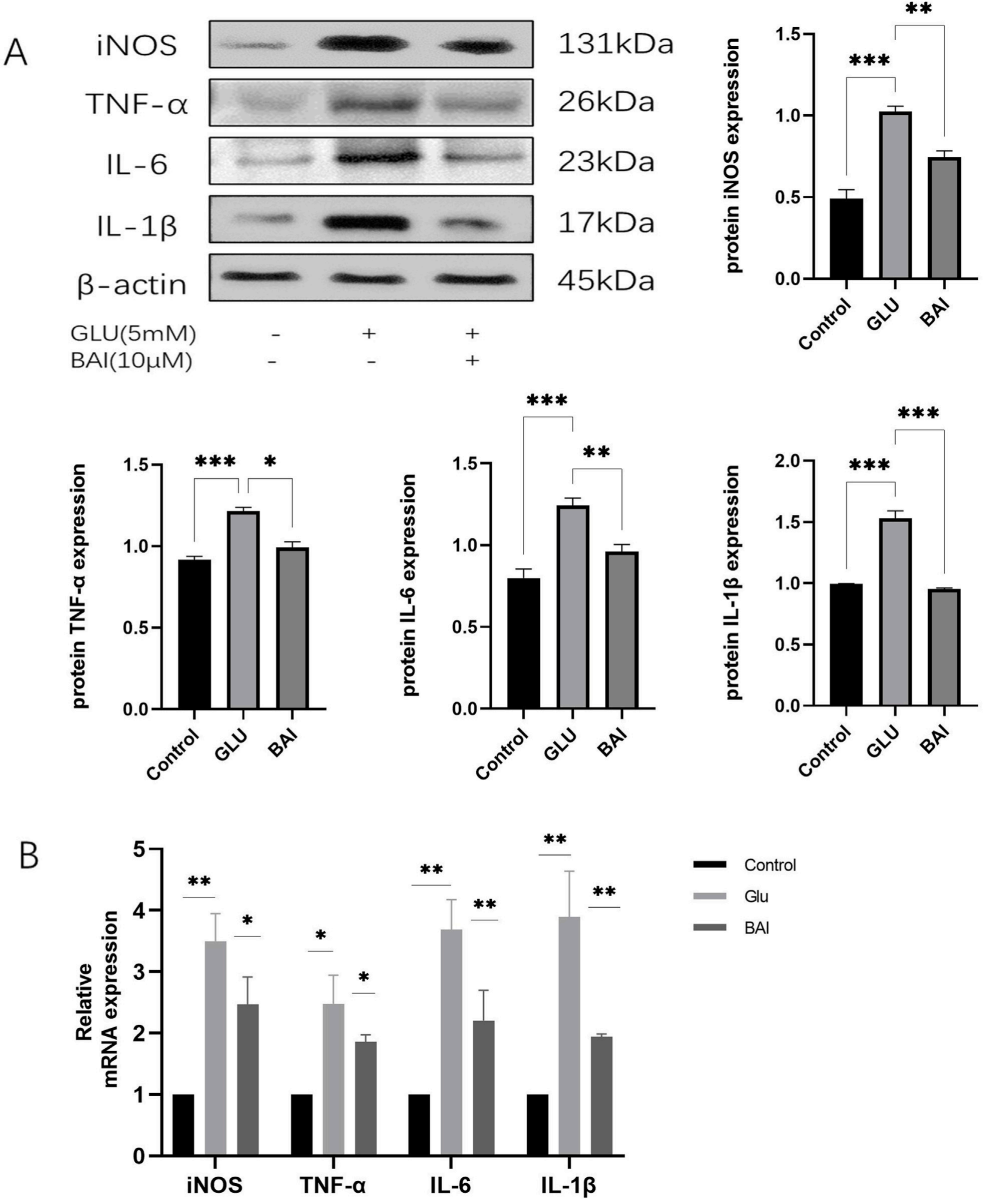
Baicalin inhibits glutamate-induced inflammatory response. **(A)** Western blot analysis was performed to detect the expression levels of inflammatory factors (inducible nitric oxide synthase (iNOS), tumor necrosis factor-α (TNF-α), interleukin-6 (IL-6), and interleukin-1β (IL-1β)). **(B)** The expression levels of inflammatory factors (iNOS, TNF-α, IL-6, IL-1β) were detected by qRT-PCR. Data represent the mean ± SEM of three independent experiments. *, *p <* 0.05; **, *p <* 0.01; ***, *p <* 0.001 indicate significant differences, and *ns* > 0.05 means no significance difference.

Previous experimental results have demonstrated a significant protective effect of 10 μM baicalin against glutamate-induced injury. Building upon this discovery, Western blot and qRT-PCR analyses were employed to assess the levels of inflammatory factors in the experiment, aiming to further investigate the potential effects of baicalin on the inflammatory response. It is well-documented that glutamate-induced oxidative stress damage can trigger the production of numerous inflammatory factors, thereby exacerbating neuronal cell death (Wang et al., 2022; Ahmad and Ahsan, 2020). The elevation of ROS has been shown to activate NF-κB, leading to the generation of inflammatory factors such as iNOS, TNF-α, IL-6, and IL-1β (Li et al., 2022). As depicted in Figure 4, the protein levels of iNOS, TNF-α, IL-6, and IL-1β (Figure 4A, *p* = 0.0029, *p* = 0.0038, *p* = 0.0027, *p* = 0.0008) along with their corresponding mRNA levels (Figure 4B, *p* = 0.043, *p* = 0.0473, *p* = 0.0052, *p* = 0.0078) significantly increased under the influence of glutamate, indicating an intensified inflammatory response. However, following baicalin treatment, the expression levels of these inflammatory factors were notably reduced, suggesting that baicalin could inhibit the inflammatory response.

### 3.5 Baicalin alleviates RGCs damage induced by glutamate in rats

**FIGURE 5:**
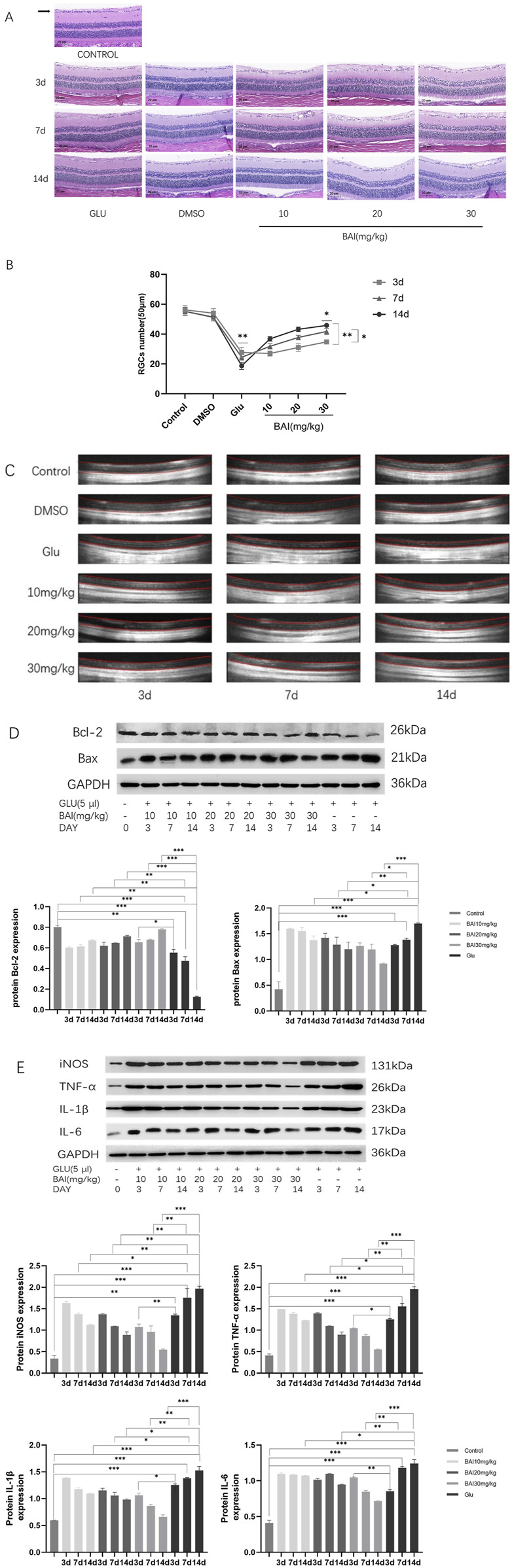
Baicalin mitigated glutamate-induced damage to retinal ganglion cells (RGCs) in rats. **(A)** Representative HE staining images of the eyeballs of rats in each group on the third, seventh, and 14th days. **(B)** RGCs count. HE staining representative images and RGCs count of eyeballs at days 3, 7, and 14. **(C)** Optical coherence tomography (OCT) was used to measure retinal thickness in rats. **(D)** Western blot analysis of apoptosis-related proteins (Bcl-2, Bax) and glyceraldehyde 3-phosphate dehydrogenase (GAPDH) expression (n = 4). **(E)** Western blot analysis was performed to detect the expression of inflammatory factors (iNOS, TNF-α, IL-6, and IL-1β) and GAPDH (n = 4). Compared with the control group. Data represent the mean ± SEM of three independent experiments. *, *p <* 0.05; **, *p <* 0.01; ***, *p <* 0.001 indicate significant differences, and *ns* > 0.05 means no significance difference.

After administration on the third, seventh, and 14th days, HE staining was employed to evaluate the pathological changes in retinal tissues across all groups (control, glutamate injury, DMSO, and baicalin) (Figure 5A). In the control group, retinal layers were distinct, and RGCs were densely and neatly arranged. In contrast, the glutamate injury group exhibited disrupted retinal tissue structure and morphology, characterized by indistinct retinal layers, diminished thickness of the retinal ganglion cell layer (GCL), and reduced RGCs density (Figure 5B). The number of RGCs was significantly lower in the glutamate-injured group compared to the control group (*p* = 0.004), confirming the successful establishment of a rat glaucoma model. Caudal vein injection of the compound mitigated these pathological changes in the retinal tissues of rats to assess the impact of baicalin and mitigate these pathological changes in the retinal tissues of rats compared to the glutamate injury group. Additionally, both GCL thickness and RGCs density were increased in the baicalin group (*p <* 0.001). These results indicate a protective effect of baicalin against glutamate-induced retinal injury.

OCT examinations were conducted to further explore baicalin’s protective effects on the retina and observe retinal changes in rats. Figure 5C and Table 2 indicated that relative to the regular control group, the full layer thickness of the retina decreased progressively on the third, seventh, and 14th days in the retinal injury group, reflecting ongoing damage. However, following baicalin treatment at various concentrations, the decreasing trend in the full layer thickness of the rat retina was mitigated to some extent, showing both time-dependent and dose-dependent effects. This suggests that baicalin exerts a protective influence on retinal structural damage in rats. In conclusion, baicalin alleviates the pathological changes induced by glutamate in glaucomatous rats and mitigates the loss of RGCs.

**TABLE 2:** Retina thickness in different rat groups.

Time	Day 3	Day 7	Day 14
Control group	193.43 ± 4.98	193.20 ± 3.39	192.09 ± 5.65
DMSO group	193.63 ± 5.41^#^	192.60 ± 6.22^#^	194.06 ± 6.20^#^
Glutamate injury group	168.84 ± 3.11^#^	154.40 ± 3.50^#▲^	129.04 ± 5.70^#▲^
Baicalin group (10 mg/kg)	171.80 ± 3.96^*^	169.80 ± 4.86^*▲^	160.20 ± 2.07^*▲^
Baicalin group (20 mg/kg)	176.63 ± 3.78^*^	174.40 ± 3.78^*▲^	166.00 ± 4.30^*▲▪^
Baicalin group (30 mg/kg)	180.21 ± 6.97^*^	178.20 ± 4.32^*▲^	175.60 ± 2.40^*▲▪^
*F*	21.021	38.338	35.051
*P*	<0.001	<0.001	<0.001

DMSO: dimethyl sulfoxide.

^#^ *P <* 0.05 vs. Control.

^*^ *P <* 0.05 vs. Glutamate injury group.

^▲^ *P <* 0.05 vs. Day3, ^▪^ *P <* 0.05 vs. Baicalin group (10 mg/kg).

On the third, seventh, and 14th days post-baicalin administration, retinal tissues from rats were collected, and the expression levels of the anti-apoptotic protein BCL-2 and pro-apoptotic protein Bax (Figure 5D), along with inflammatory factors (iNOS, TNF-α, IL-6, IL-1β), were assessed through Western blot analysis (Figure 5E). This analysis aimed to further explore the protective effects of baicalin on glutamate-induced retinal injury. The results showed that glutamate treatment upregulated the expression level of Bax (*p <* 0.05) but downregulated the expression level of Bcl-2 (*p <* 0.05). Compared with the control group, the expression level of inflammatory cytokines increased in a time-dependent manner (*p <* 0.05). After baicalin treatment, the expression of these proteins was significantly reversed in a dose- and time-dependent manner.

### 3.6 Protective effect of baicalin on RGCs is related to the JAK/STAT signaling pathway

**FIGURE 6:**
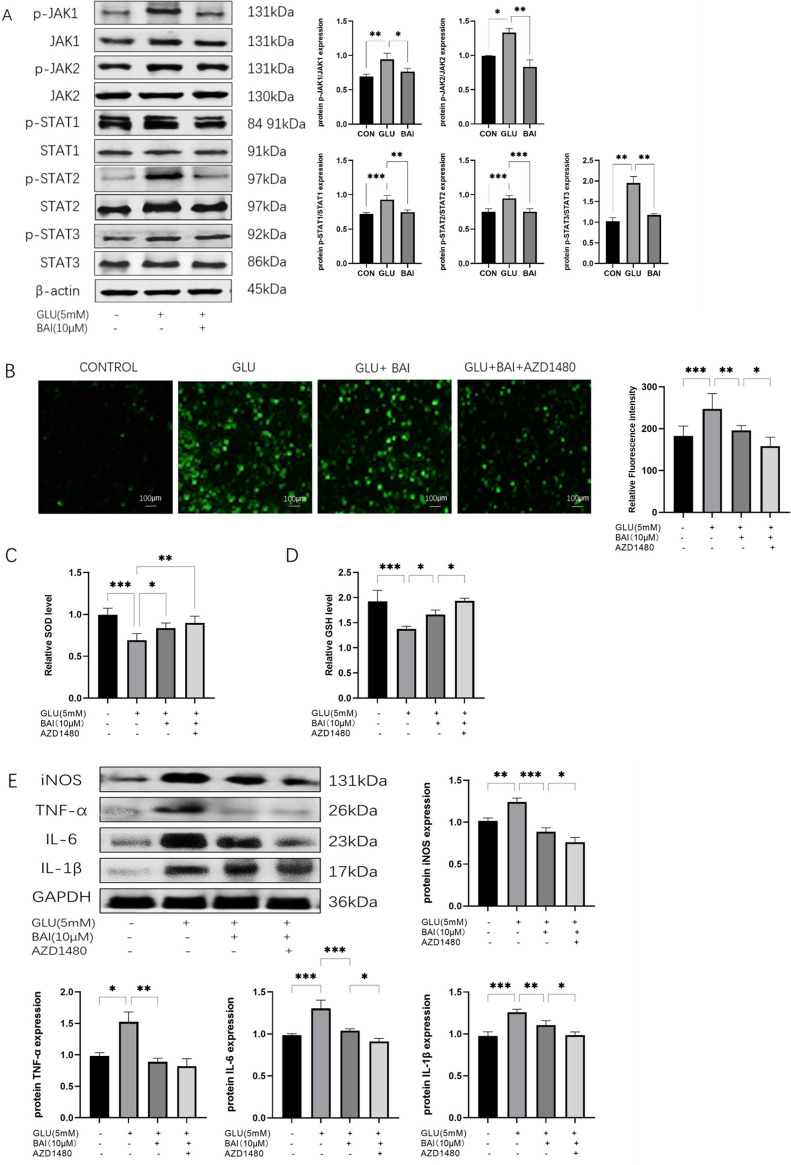
The correlation between the protective effect of baicalin on RGCs and the JAK/STAT signaling pathway. **(A)** Western blot analysis of P-JAK1, JAK1, P-JAK2, JAK2, p-STAT1, STAT1, p-STAT2, STAT2, P-STAT3, STAT3, and β-actin expression. **(B)** Analysis of ROS fluorescence images. **(C, D)** Levels of ROS, SOD, and GSH in each group (normal control group, glutamate injury group, glutamate + baicalin group, glutamate + baicalin + JAK/STAT inhibitor group). **(E)** Western blot analysis detected the protein expression levels of inflammatory factors (iNOS, TNF-α, IL-6, IL-1β), with GAPDH as the internal reference (n = 3). Data represent the mean ± SEM of three independent experiments. *, *p <* 0.05; **, *p <* 0.01; ***, *p <* 0.001 indicate significant differences, and *ns* > 0.05 means no significance difference.

Through the assessment of protein expression levels in the JAK/STAT signaling pathway, it was observed that the phosphorylation levels of JAK and STAT in the baicalin-treated group were significantly lower than those in the control group (Figure 6A, *p* = 0.0078, *p* = 0.0259, *p* = 0.0006, *p* = 0.0004, *p* = 0.0052). This finding suggests that baicalin effectively suppresses the activation of the JAK/STAT signaling pathway, potentially influencing associated cell signaling pathways and contributing to neuroprotection. Subsequent experimental results confirmed that baicalin treatment led to a significant reduction in the phosphorylation levels of JAK and STAT (*p* = 0.045, *p* = 0.0087, *p* = 0.0014, *p* = 0.0004, *p* = 0.0087), further supporting the inhibitory role of baicalin on the JAK/STAT signaling pathway.

The results demonstrated that in the glutamate + baicalin group, the ROS level was lower compared to the glutamate injury group, indicating that baicalin exhibited a specific antioxidant effect. Subsequently, upon the addition of JAK/STAT inhibitors, a further decrease in ROS level was observed (Figure 6B, *p* = 0.0483), accompanied by an increase in SOD and GSH levels (Figures 6C–E, *P* = 0.0032, *p* = 0.0416). The antioxidant activity of baicalin was enhanced due to the JAK/STAT inhibitor AZD1480. Furthermore, a reduction in the expression levels of inflammatory factors was observed after treatment with glutamate + baicalin + JAK/STAT inhibitor, as opposed to the baicalin group (Figure 6F, *p* = 0.0442, *p* = 0.057, *p* = 0.0478, *p* = 0.0119), suggesting that the JAK/STAT inhibitor augmented the anti-inflammatory effect of baicalin and further bolstered its neuroprotective properties.

## 4 Discussion

Glutamate, the primary excitatory neurotransmitter in the central nervous system, is closely associated with the onset and progression of various retinal diseases, such as glaucoma, diabetic retinopathy, optic neuritis, and ischemic optic neuropathy (Liu and Prokosch, 2021). Excessive glutamate levels can lead to cellular and retinal damage through oxidative stress and inflammation. Finding drugs that can inhibit or reverse glutamate excitotoxicity and investigating their mechanisms of action are essential goals in preventing and treating retinal diseases. Glutamate-induced RGCs death is an *in vitro* model that studies neurodegenerative diseases and drug protective effects (Ma et al., 2019; Dai et al., 2022). Therefore, our study focused on R28 cells and examined the effect of glutamate on R28 cell injury. Different concentrations of glutamate (1 mM, 3 mM, 5 mM, 10 mM) were used to induce RGCs damage, with the results indicating that 5 mM glutamate reduced RGCs survival rates. Consequently, our study aimed to investigate whether baicalin could inhibit glutamate-induced excitotoxicity, thereby protecting RGCs from oxidative stress damage.

Numerous studies have demonstrated the pharmacological effects of baicalin, including anti-inflammatory, antioxidant, and anti-apoptotic properties, with protective effects observed in various cell types (Zhu et al., 2020; Fan et al., 2021; Sui et al., 2021). Baicalin has been shown to promote neural differentiation of neural stem/progenitor cells and alleviate neuroinflammation (Li et al., 2020; Li et al., 2012). Network pharmacology analysis and animal experiments have revealed that baicalin effectively improves pathological changes in the optic nerve fiber layer in glaucoma (Yang et al., 2021). Therefore, in this experiment, R28 cells injured by glutamate were treated with different concentrations of baicalin for 24 h and 48 h. The results showed that the protective effect of 10 μM baicalin was more significant, while the protective effect decreased with the increase of baicalin concentration. Similarly, Lei (Gong and Zhu, 2018) et al. found that when baicalin was applied to human trabecular meshwork cells, the cell viability did not decrease significantly at a concentration of 15 μM, whereas the number of viable cells decreased significantly at concentrations of 20 μM and 50 μM. However, the mechanism needs to be further investigated. Whether it is due to the cytotoxicity or the antioxidant effect of baicalin interfering with the intracellular redox balance at high concentrations, it results in cellular oxidative stress response and decreased cell viability. *In vivo,* experimental results showed that baicalin alleviated pathological changes induced by glutamate in rats with glaucoma and inhibited RGCs apoptosis. Furthermore, its protective effect became more pronounced with increasing therapeutic time and concentration. Additionally, different drug delivery methods may affect the efficacy and pharmacokinetics of baicalin. For instance, intravenous injection can rapidly increase blood drug concentration and is suitable for acute inflammation requiring rapid symptom control, while local drug delivery can reduce drug distribution in the body and minimize toxic side effects. Therefore, further exploration of baicalin’s concentration range and delivery methods *in vivo* is necessary to determine the optimal concentration and delivery method for treating RGCs injury.

Recently, the JAK/STAT signaling pathway has gained attention in the pathogenesis and treatment of various diseases. This pathway is crucial in mediating physiological and pathological processes such as oxidative stress, inflammatory response, and neuronal apoptosis (Yang et al., 2021; Paithankar et al., 2021). Baicalin alleviates myocardial ischemia-reperfusion injury and inflammation caused by retinal ischemia-reperfusion injury in rats by inhibiting the phosphorylation levels of the JAK2/STAT3 signaling pathway (Dong et al., 2021). IL-6, the primary activator of the JAK/STAT signaling pathway, has been detected in the aqueous humor of glaucoma patients (Xu et al., 2020). We investigated whether baicalin protects retinal ganglia by regulating the JAK/STAT signaling pathway. The results showed that baicalin reduced the phosphorylation levels of JAK and STAT, thereby reducing oxidative stress and inflammatory factor levels and alleviating glutamate-induced injury. OCT measurements of retinal thickness in rats revealed a significant decrease in the glutamate group, which was mitigated by baicalin. These findings provide essential theoretical and experimental foundations for further exploring the role of the JAK/STAT signaling pathway in disease incidence, development, and the development of related drugs. Future research can further investigate the mechanisms of the JAK/STAT signaling pathway in different disease models to provide new targets and strategies for treating related diseases.

In conclusion, baicalin exhibits potential neuroprotective effects and promising application prospects, but its drug delivery and utilization in the eye require further research and exploration. With the advancement of modern drug delivery technology, new delivery methods for baicalin, such as nanocarrier technology and microsphere technology, are constantly being explored. These technologies can improve drug bioavailability, prolong drug half-life in the body, reduce toxic side effects, and enhance drug efficacy and safety. Additionally, due to the lack of effective protective drugs for the optic nerve, no positive control group was included in this study. Therefore, future research should expand the concentration range of baicalin *in vivo* to determine its optimal concentration for treating RGCs injury.

## 5 Summary

Baicalin protects against glutamate-induced oxidative stress injury in RGCs, effectively reducing oxidative stress and inflammation, decreasing apoptosis, and improving the pathological changes in a rat model of RGCs injury. It also reduces RGCs death, providing a basis for further exploration of its mechanisms. Baicalin inhibited the JAK/STAT signaling pathway, protecting RGCs from oxidative stress. These findings provide an experimental basis for the potential application of baicalin in treating RGCs injury.

## Data Availability

The original contributions presented in the study are included in the article/Supplementary Material, further inquiries can be directed to the corresponding authors.
