# Supplementary figures and images for: Protective effect of baicalin on oxidative stress injury in retinal ganglion cells through the JAK/STAT signaling pathway in vitro and in vivo
Source: Front Pharmacol. 2024 Oct 31;15:1443472. doi: 10.3389/fphar.2024.1443472 (PMC11565601; doi:10.3389/fphar.2024.1443472)

细胞 WB

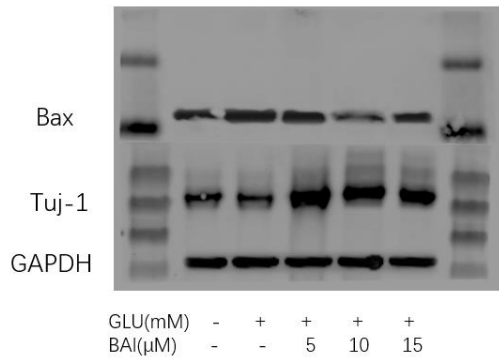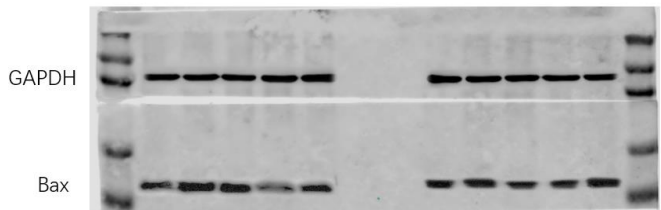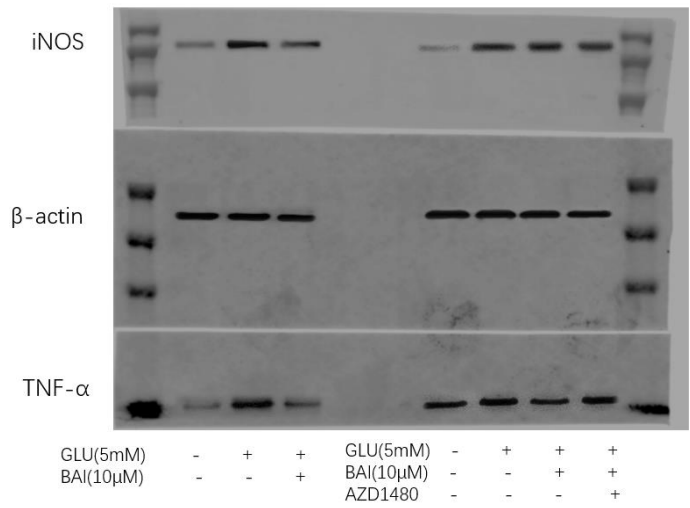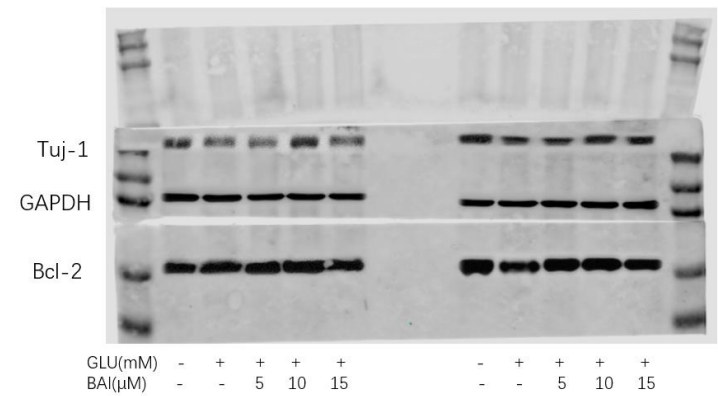

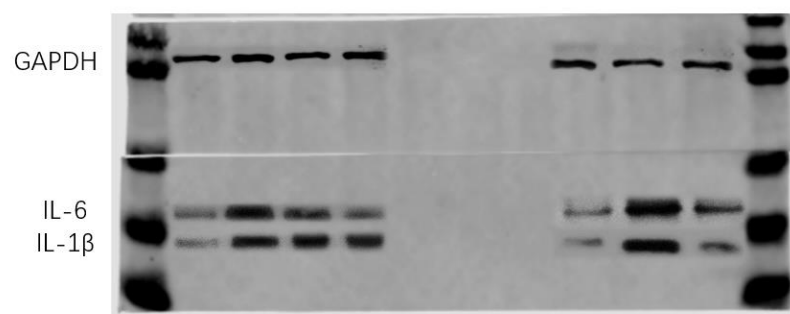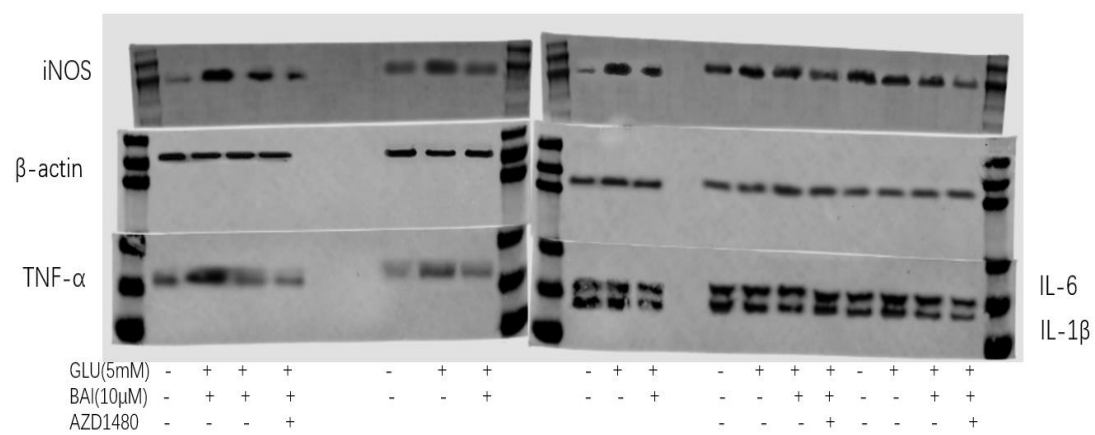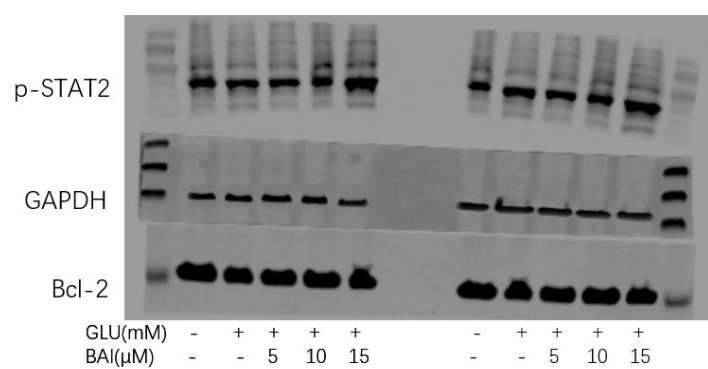

# JAK/STAT 条带 (分组: Contrul , Glu, Bai)

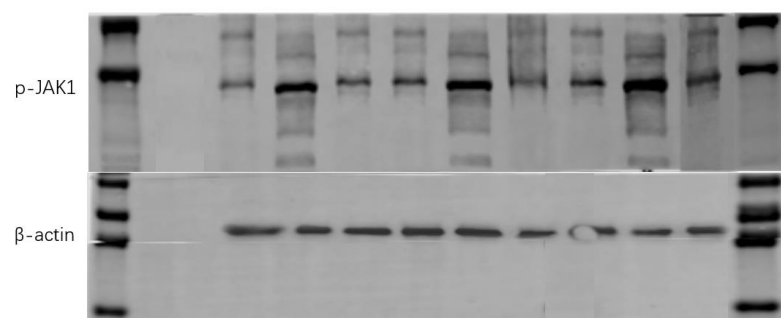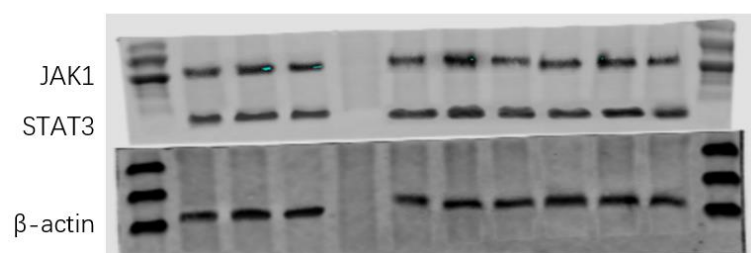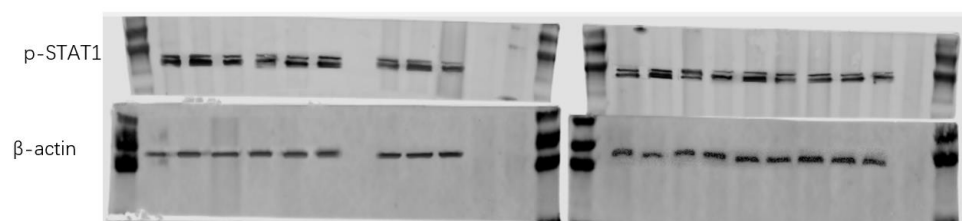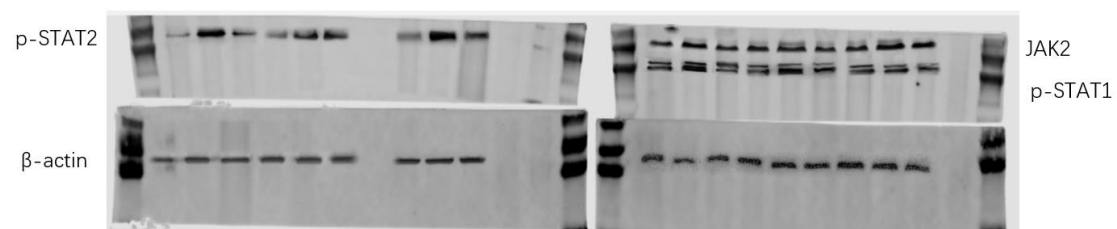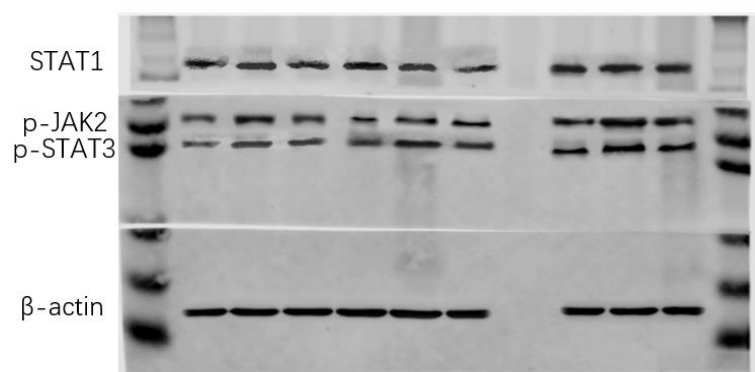

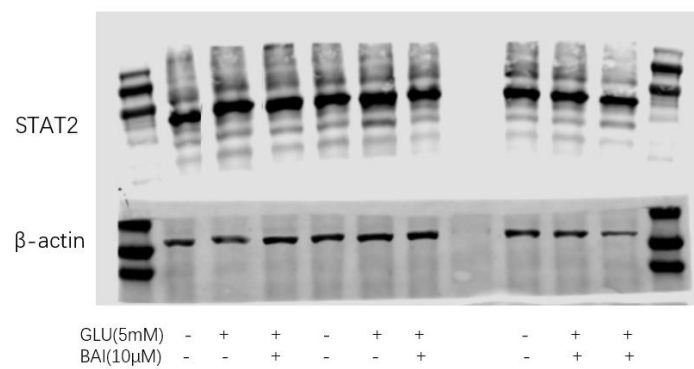

## 动物 WB

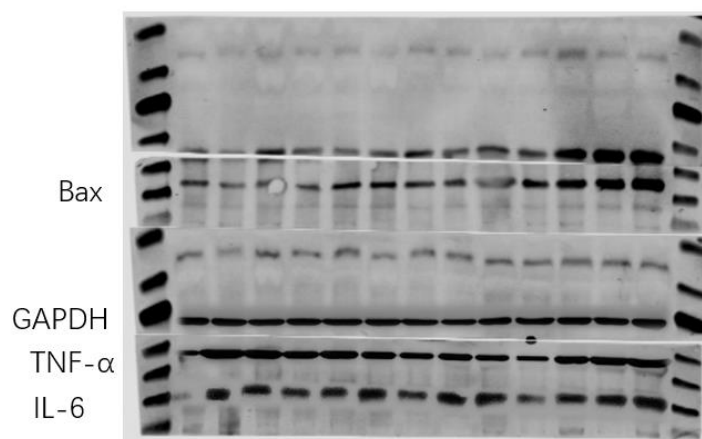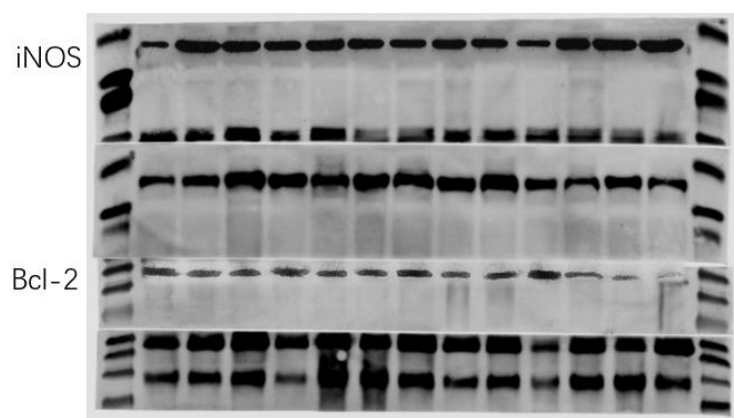

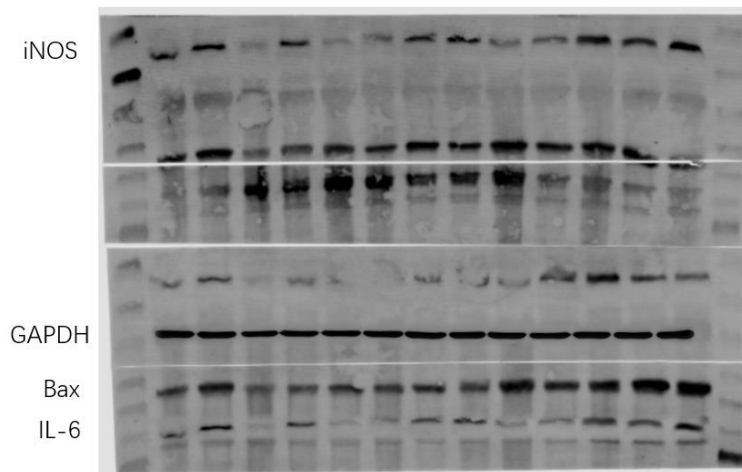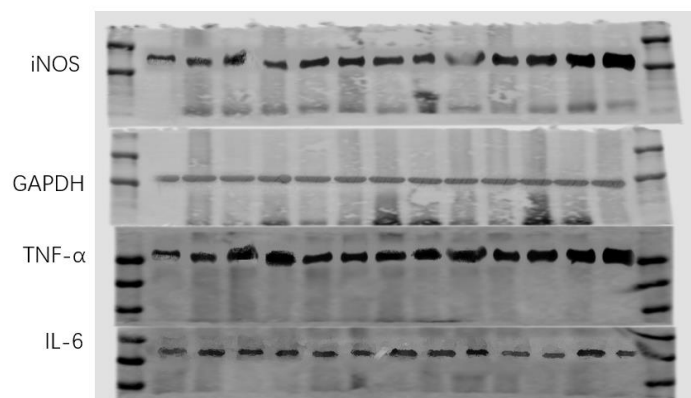

Supplement: Supplementary file 1 [file DataSheet1.PDF]
